# Extending the temporal context of ethnobotanical databases: the case study of the Campania region (southern Italy)

**DOI:** 10.1186/1746-4269-5-7

**Published:** 2009-02-19

**Authors:** Antonino De Natale, Gianni Boris Pezzatti, Antonino Pollio

**Affiliations:** 1 Department Ar.Bo.Pa.Ve, University of Naples "Federico II" – Via Università, 100, 80055 Portici (NA), Italy; 2 WSL Swiss Federal Institute for Forest, Snow and Landscape Research, Ecosystem Boundaries Research Unit – Via Belsoggiorno 22, CH-6500 Bellinzona, Switzerland; 3 Department of Biological Sciences/Section of Plant Biology, University of Naples "Federico II" – Via Foria, 223, 80139 Napoli, Italy

## Abstract

**Background:**

Ethnobotanical studies generally describe the traditional knowledge of a territory according to a "hic et nunc" principle. The need of approaching this field also embedding historical data has been frequently acknowledged. With their long history of civilization some regions of the Mediterranean basin seem to be particularly suited for an historical approach to be adopted. Campania, a region of southern Italy, has been selected for a database implementation containing present and past information on plant uses.

**Methods:**

A relational database has been built on the basis of information gathered from different historical sources, including diaries, travel accounts, and treatises on medicinal plants, written by explorers, botanists, physicians, who travelled in Campania during the last three centuries. Moreover, ethnobotanical uses described in historical herbal collections and in Ancient and Medieval texts from the Mediterranean Region have been included in the database.

**Results:**

1672 different uses, ranging from medicinal, to alimentary, ceremonial, veterinary, have been recorded for 474 species listed in the data base. Information is not uniformly spread over the Campanian territory; Sannio being the most studied geographical area and Cilento the least one. About 50 plants have been continuously used in the last three centuries in the cure of the same affections. A comparison with the uses reported for the same species in Ancient treatises shows that the origin of present ethnomedicine from old learned medical doctrines needs a case-by-case confirmation.

**Conclusion:**

The database is flexible enough to represent a useful tool for researchers who need to store and compare present and previous ethnobotanical uses from Mediterranean Countries.

## Background

In the last decade, several information systems have been developed to collect and manage the traditional knowledge (TK) on the use of plants from different geographical areas. As far as the Mediterranean basin is concerned, networks like MEDUSA [1] have been established to identify native and naturalized plants, and to set up an on-line database. In the same area the integrated and multi-disciplinar project RUBIA [2] focuses on the collection and dissemination of TK related to uses of wild and neglected cultivated plants, also exploring the relationships to folkloric history and socio-cultural context. On a wider geographical scale, Thomas et al. [3] developed an electronic archive based on object database technology for submission, storage and retrieval of ethnomedicinal data, and Skoczen and Bussmann [4] have recently presented their International Ethnobotany Database (ebDB), an on-line database, which provides a generic solution for ethnobotanical data storage, being multilingual, and open to anyone wants to add information. Although those databases implement advanced solutions regarding linguistic and semantic issues [3], they have not been designed for the storage of data ranging over large temporal extensions, and, at present, no data base repository has been specifically developed with a focus on historical ethnobotany.

**Figure 1:**
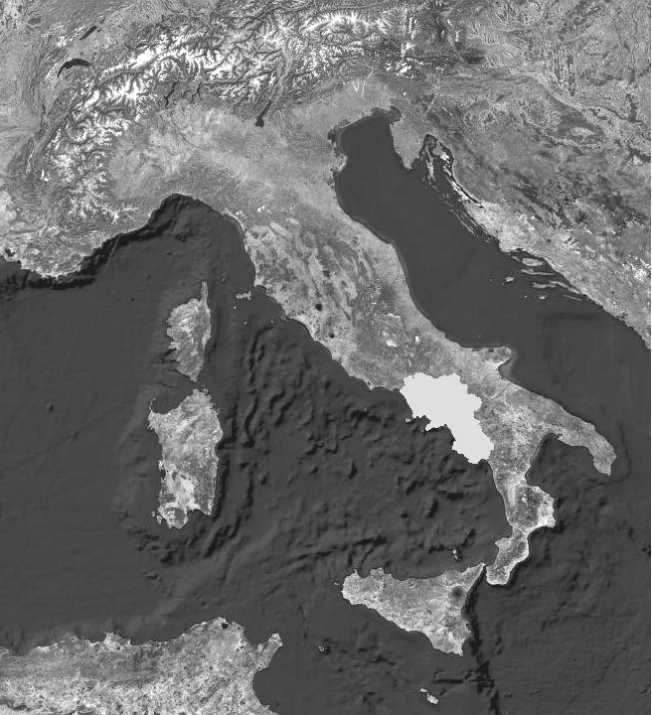
**Location of Campania in Italy**.

It has been recently stressed that one of the unresolved problems of the present ethnobotanical and ethnopharmacological studies is the limited contribution of disciplines like anthropology and historical sciences [5,6]. As a consequence the focus of ethnobotanical research is rarely given by the comprehension of the traditional health systems and their development during the centuries. On the other hand, the complexity of this approach requires adequate tools to manage the complex flow of information collected. The historical complexity, common to Mediterranean area [7], requires an integrated approach to understand the context and the evolution of local traditional knowledge on plant uses [8,9]. As a case-study, we selected the Campania region (southern Italy, Figure 1), which has been inhabited since prehistoric times by different ethnic groups, thus merging different traditions into a unique *corpus *of traditional knowledge. Before the Roman colonization, Greek and Etrurian colonies occupied this region, together with indigenous Italic people, as Osci and Samnites [10]. Later on, the Region of Campania was annexed by the Romans and, after the fall of the Western Roman Empire, different populations ranging from Goths and Byzantines, to Arabs, Normans and Longobards occupied part of the territory or established lasting relationships with local populations.

A relational database has been elaborated with the goal of providing a standard for the storage of historical series of ethnobotanical data in the Mediterranean region, allowing the temporal exploration of links and meanings.

## Methods

### Development of the database

The database prototype has been implemented as a local relational database. Different implementation solutions were tested, according to the requirements and use cases. Present and ancient ethnobotanical data were organized in the same table structure, allowing some costs of redundancy in favor of the homogenization of data and queries. Because flexibility was a major issue for our archive, object oriented concepts were implemented, such as unlimited hierarchical structures (self-joined tables), particularly for categorized look-up tables and species taxonomy, and inheritance concepts, allowing e.g. information sources to be a reference, a person, an herbal or an archeological remain. Those techniques allowed us to develop a minimal data structure fitting different kind of information, and to perform queries at different levels of detail.

**Figure 2:**
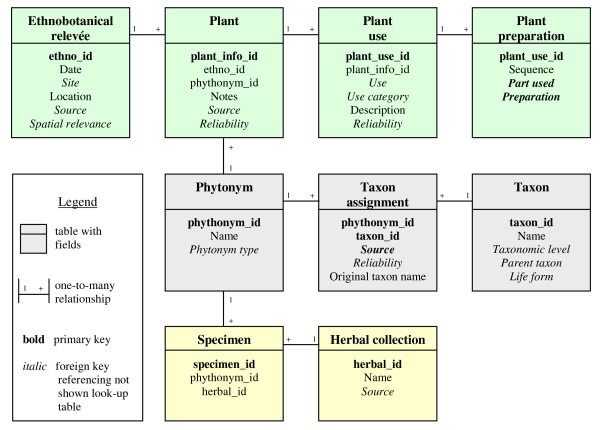
**Database structure**. Simplified structure of the database, showing the main tables and the relationships between them. The tables in green are related to ethnobotanical information, in grey to plant names management and in yellow to specimen and herbal collections.

The structure of the developed repository is summarized in Figure 2 and in Table 1.

**Table 1:** Short description of the tables of the database (in italic are listed look-up tables not shown in Figure 2).

**Table name**	**Description**
**Ethnobotanical relevée**	Details of the ethnobotanical relevée, like site, coordinates, source (e.g. collector) and spatial relevance. This relevée can then contain information on multiple plants.
**Plant**	Information relative to the plant, like reference to the phytonym, source (e.g. informant), reliability of the phytonym identification and general notes. A plant can then contain details on multiple uses.
**Plant use**	Information on the use of the plant, namely classified use and use category, use description and reliability (especially for information from the ancient treatises or archeological remains).
**Plant preparation**	The preparation of the plant is stored according to preparation mode and plant part used. Multiple steps for the preparation of a single plant use (receipt) can also be recorded.
**Phytonym**	Different phytonyms, of different types. In this table are stored also the scientific names, including synonyms.
**Taxon assignment**	Join table storing the taxon assignments to the phytonyms, with the source and the reliability. For the case of scientific phytonyms, and for most of the Italian phytonyms, the reliability is considered sure. A phytonym can have multiple putative identifications.
**Taxon**	Scientific name of the taxon, organized in a hierarchical structure (at different taxonomic levels), e.g. Labiatae > Thymus > Thymus serpyllum. Only the accepted scientific synonym is stored in this table.
**Specimen**	Herbal specimen information.
**Herbal collection**	Details on the herbal collection.
*Life form*	Different life forms, organized in a hierarchical structure (with different detail levels), e.g. Therophytes > erect.
*Phytonym type*	Ancient, Italian, popular and scientific names.
*Plant part*	Different plant parts, organized in a hierarchical structure (with different detail levels), e.g. aerial part > leaf > young leaf.
*Preparation*	Different preparation steps, organized in a hierarchical structure (with different detail levels), e.g. cooked > boiled, fried.
*Reliability*	Sure, nearly sure, probable, presumed, doubtful, wrong.
*Site*	Different sites, organized in a hierarchical structure (at different spatial scales), e.g. Mediterranean > Italy > South Italy > Campania > Napoli.
*Source*	Source description, can be of different types (bibliographic reference, collector, informant, herbal, archeological). According to the referencing tables, only subsets of this table can be selected.
*Spatial relevance*	Point, local, municipality, region, province, nation.
*Taxonomic level*	Family, genus, species, subspecies.
*Use*	Different plant uses, organized in a hierarchical structure (with different detail levels), including medicinal (e.g. health > neurological disease > headache), ceremonial, alimentary, domestic and veterinary uses.
*Use category*	Ancient medicine, official medicine, popular, ceremonial

The archive currently allows the storage and management of following information:

• ethnobotanical information from interviews (informants), historical herbarium specimens and documents (recent, historic and ancient sources), according to a hierarchical catalogue of plant uses and preparations (medicinal, alimentary, ceremonial, veterinary, domestic). It is also possible to include ethnobotanical information from archeological remains, although the relationships to the relative databases have not yet been implemented.

• plant names information: plant popular names (local or ancient), and their relationship to species taxonomy [11]. Information on reliability of species identification (also multiple options are possible, according to hypothesis of different authors);

• information on specimens in historical herbals.

Ethnobotanical data were organized in a relational structure: a relevée can group information on multiple plants, which can be used for different scopes, each being reached with one or a sequence of preparation steps. Traditional knowledge cannot be easily categorized and therapeutic uses are often intermingled with ceremonial and symbolic ones. Since attempts to categorize these kinds of uses are not always meaningful, the storage of a description that reports in detail a specific application of a plant is recommended. However, to allow a more effective search within the database, beliefs and practices dealing with folk uses of a species were grouped in use categories, ranging over medicinal (including affection and mode of administration of the plant), ceremonial (symbolic, ritual, religious including any belief concerning the plant), alimentary, domestic (from the control of undesired animal in the house, to house construction, or wooden utensils), veterinary uses. These categories have been organized in a hierarchical structure and may be assigned according to the detail level of the information gathered. A reliability degree of those use assignments is given, particularly for data from the ancient treatises and from archeological remains. The principle of storing information with different levels of accuracy has been implemented also for the preparation steps (mechanical breaking, cooking, drying, ...) and for the plant parts involved (above ground part, bark, leaf, young leaf, below ground part, root, bulb, tuber, ...).

This level of semantic detail allows fine tuned query possibilities and is needed if a future improvement to a multilingual application is planned.

The database is intended to store and manage data from pre-Linnean times to present. At the moment the oldest entries stored in the database, dealing with plant uses in Mediterranean region during Antiquity, come from studies carried out in the last decade on *Corpus Hippocraticum *[12] treatises, Dioscorides "*De Materia Medica*" [13-15], Pliny's *Naturalis Historia *[16], and Pseudo-Apuleius *Herbarium *[17]. Since ancient texts provides no iconography and limited description of plants; the identity of these species is always a matter of controversial [18]. For example, according to Buenz [19], the identification of the plants quoted in the *Corpus Hippocraticum *is a largely overcome challenge, whereas Raven [20] is clearly skeptical about the possibility of resolving the identity of any Greek phytonym down to the species level, in accordance with Linnaean nomenclature. In order to explicitly implement in our database this uncertainty, a join table with the putative identifications of ancient phytonyms with one or more Mediterranean species has been implemented with the proposed identifications according to Aliotta et al. [12] and Stirling [21]. Thus this allows a critical evaluation of ancient traditional knowledge about the uses of a plant. We used this specific structure to enable us also to manage synonym relationships of scientific names or identical phythonyms referring to different species, according to regions or authors. In this respect, our phythonym table contains popular, ancient, Italian and scientific names, allowing a maximum flexibility when entering new ethnobotanical records.

Since a consistent number of our entries come from plant labels in historical herbals, also detailed information on specimen and herbal collections was stored in the database.

Information sources can be of multiple types, namely bibliographic references, collectors, informants, historical herbals or archeological remains. All or subsets of these types can be referenced, according to the tables where a source information is needed.

As already mentioned, reliability of the information stored in the database is handled at multiple levels, namely: phytonym identification (Plant table), which in the case of informants or collectors is useful, tentative identifications (Taxon assignment table), used mainly for data from the ancient treatises, and use assignments (Plant use table), for data from ancient sources or archeological remains.

**Figure 3:**
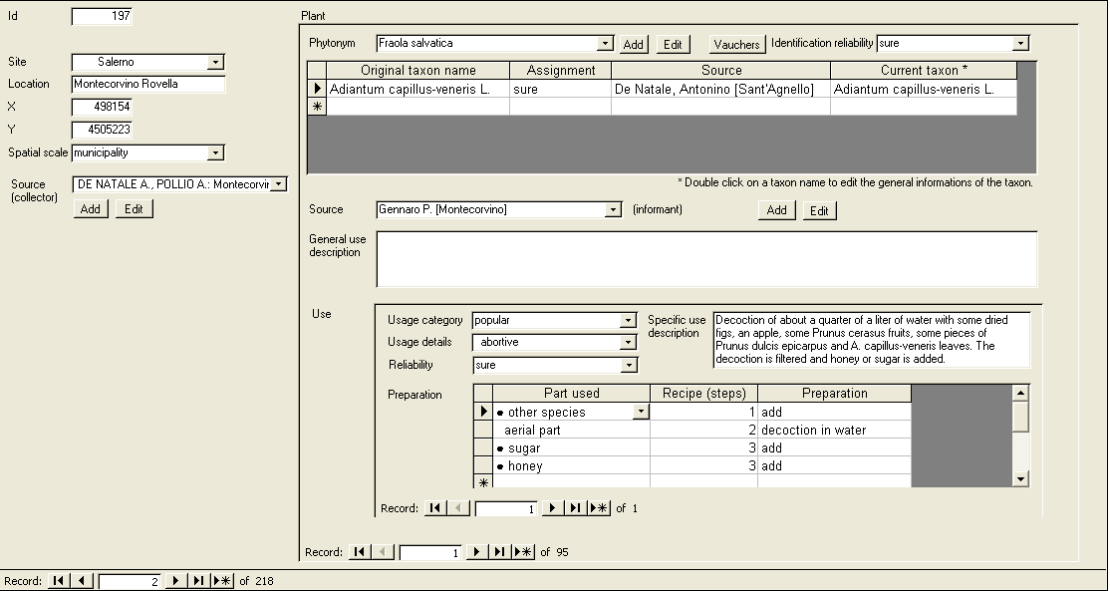
**Main data management mask, showing a record of recent ethnobotanical data**.

**Figure 4:**
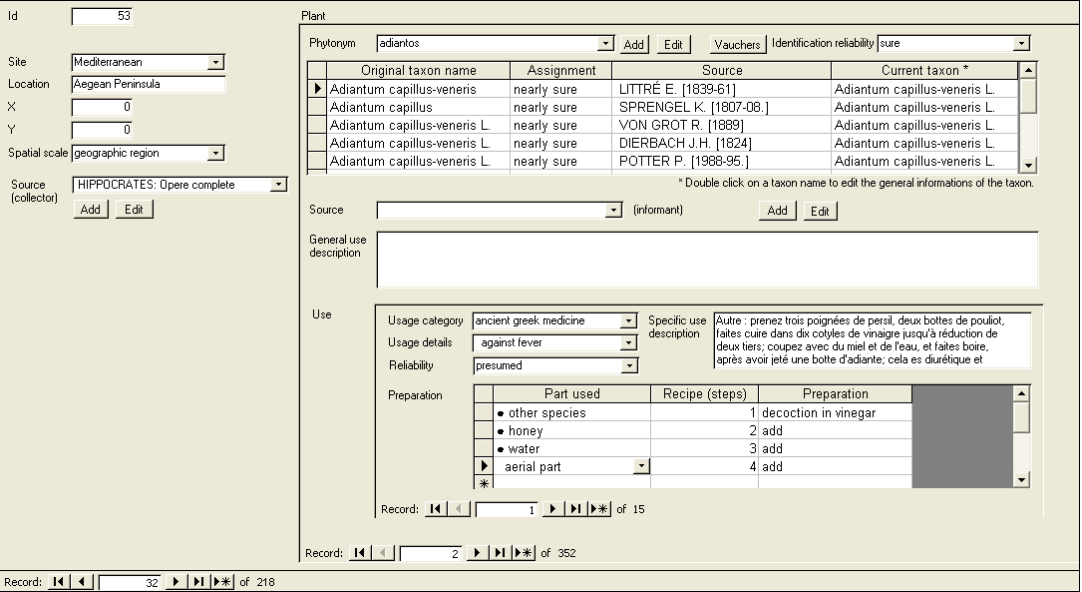
**Main data management mask, showing a record of ancient ethnobotanical data**.

Figures 3 and 4 show two screenshots of the main data management mask, respectively with recent and ancient ethnobotanical data. At this development stage, neither administration of user privileges on data subsets nor multilingual support have been implemented.

Different query features allow the user to get a temporal drawing of the importance of a species within the life of a community. A query for a species or a genus returns all the available information, either from published works, unpublished interviews, labels of historical herbals or archeological remains. The hierarchical features implemented for uses, plant parts, preparations and sites (from national to municipality level) allows both generic or fine-grained queries for those parameters, according to the requirements of the user. Particular query settings can be specified, in order to extend the queries to rougher levels of detail, e.g. querying for a genus instead of a species, thus including all the species of that genus, or querying for neurology instead of headache, thus including all neurological affections. Such latter use comparisons can only be performed in the frame of the specified hierarchical structure, which implicitly defines the degree of similarity of the uses. In the future, different hierarchical classifications of the stored uses could be defined and added to the use table, thus enhancing comparative capabilities of the archive.

The performed queries return the appropriate reliability information. This is particularly important for the case of multiple putative identifications of ancient phytonyms, where a query for a given use produces multiple report lines for the same ethnobotanical information, according to the taxon assignments. The tuning of reliability settings allows restricting the query from all the record combinations (for a case by case analysis) to only records with a certain reliability degree or to only record combinations with the highest reliability.

Also historical summary information can be retrieved (analyzing the highest reliabilities), matching a plant binomial with a specific use, and showing for example how many times a given medicinal use of a species is reported in the database for a defined period.

The repository has been implemented with PostgreSQL8.3 [22], while entry masks have been developed with Microsoft^® ^Access 2000 [23].

### Campania as a case study

At present time only data on flowering plants have been stored in the database, but it is possible to extend the archive also to non-vascular plants, algae and fungi. The interval of time was chosen because plant nomenclature from XVIII century onward is based on Linnean classification, which allows a more reliable approach to species identification. At the moment, all the data from modern (1950 – present days) ethnobotanical studies carried out in Campania have been stored in the database, together with the available information from different historical sources dating back from the first half of 1900's to XIX century. The majority of the records were extracted from hundreds of published works, both popular and scientific, which belong to three main types of sources:

I) floristic description of a territory, which often contained ethnobotanical information (i.e. Gussone [24]; Pasquale [25]; Pasquale and Pasquale [26]).

II) the labels of the historical herbaria samples of Herbarium Porticense (PORUN), that in same case reported some indications about the use of a plant in the site where it was collected (collection Vincenzo and Francesco Briganti of Herbarium Porticense – PORUN)

III) medical treatises of physicians of past centuries (i.e. Petagna [27]; Limoncelli [28]; Pasquale and Avellino [29]), which contains also the description of folk remedies, thus representing an important source of ethnopharmacological information.

## Results and discussion

### Data since the XVIII century

The database currently includes 474 different species, which have been used in Campania between the end of XVIII century and the 2007.

1672 different uses have been recorded for the species listed in the database, ranging from medicinal, to alimentary, ceremonial, domestic and veterinary. Table 2 represents the distribution of the number of data (including number of sources of information and number of species) between XIX and XX Century. It is not surprising that the majority of data are related to recent studies, since previously folk knowledge was often largely disregarded. Caution should be adopted to the approach of such heterogeneous material. The studies dealing with the traditional plant knowledge in Campania which have been conducted approximately from 1950s onward, have been carried out according to the procedures adopted worldwide for ethnobotanical research. On the other hand, historical data stored in the archive allow to elicit information from sources which hardly represent a random sample of a statistic population. They can be derived from a single observation or from memories of very few individuals, and are only rarely a detailed recollection of uses coming from a population. The structure of information on plant uses from past centuries precludes any quantitative approach in comparison between past and present traditional knowledge of populations living in Campania. Notwithstanding this limitation, the historical ethnobotanical archive represents a useful tool to understand cultural transformations occurring on plant uses over the generations, particularly shedding light on continuity and changes in folk phytotherapy.

**Table 2:** Number of Campanian ethnobotanical records of plants in the last two century, according to the stored datasets.

**Time (period)**	**Number of data**	**Number of information sources**	**Number of species**
**2000–2007**	333 426 *	4	162 238 *

**1950–1999**	645 242 *	27	282 138 *

**1900–1949**	103	13	60

**1850–1899**	150	9	120

**1800–1849**	59	9	49

**Total**	1958	62	558

Numbers are referred to published data; * number of unpublished data.

From the analysis of the information stored in the database, it is possible to make some generalizations on the evolution of ethnobotanical knowledge in Campania. Several trends remain unchanged over the last two centuries, such as the prevalence of data about medicinal plants, included those used in the so-called food medicine, which was often mentioned in XIX century sources. The occurrence of a relatively high number of medicinal citations is also due to the interests of the authors of that time, generally physicians or chemists.

**Figure 5:**
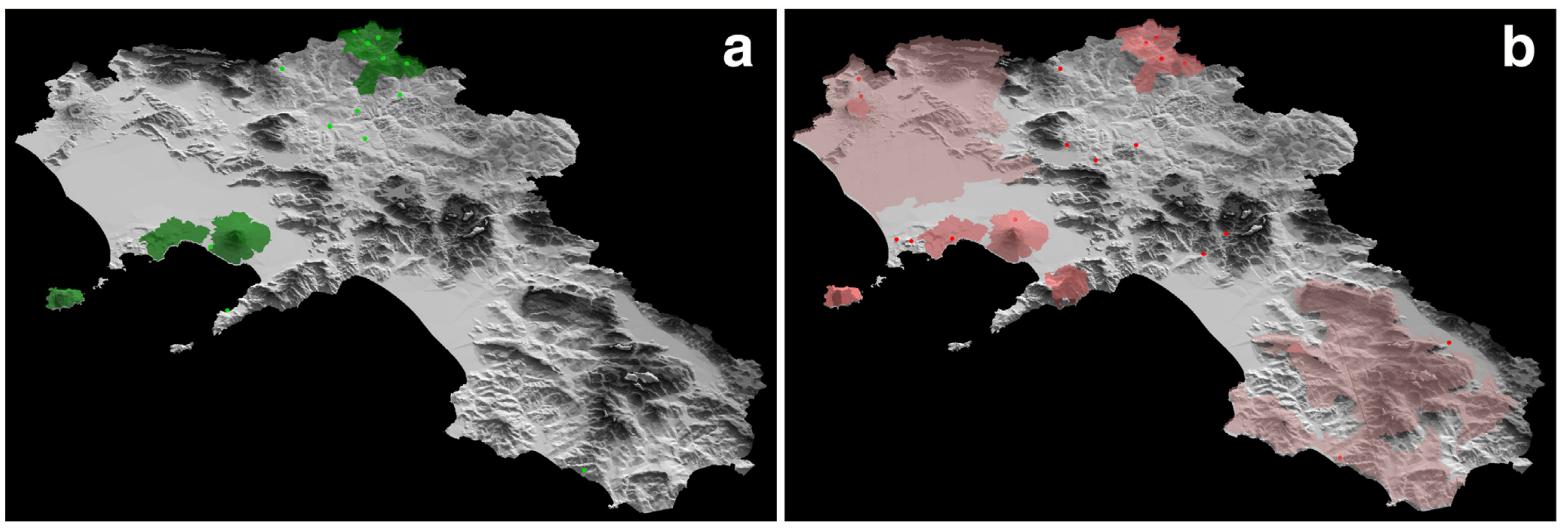
**Distribution of ethnobotanical information in Campania region during the XIX (a) and XX (b) century**. Points (red circle and green circle) designate local information (territories up to 10 k2). Colored areas are related to data generically attributed to large territories. Dark areas (green and dark pink) = territories between 10 and 100 k2, light pink areas = territories > 100 K2 ha.

The data were analyzed for both content and geographical distribution. In Figure 5a is shown the distribution of ethnobotanical information in the XIX century in Campania, and in Figure 5b that related to the XX century. Figures 5a and 5b reveal that some Campanian areas, which are ecologically homogeneous and share a common historic context, have been studied in some detail, whereas for other territories information is very scanty. In the last two centuries, a remarkable convergence of ethnobotanical studies has been carried out on Sannio, a territory which in part correspond to Samnium of Pre-Roman and Roman Age [10]. Moreover, from the comparison between figure 5a and 5b a difference clearly emerges: some studies made in the first mid of the XX century report on ethnobotanical data attributed to large macro-areas of Campania, without any further detail. In figures 5b the areas interested by this kind of studies are marked with a pink colour. Although this reports can reveal meaningful information on the value of natural resources to local people, it is not possible to relate this information to a specific community. In our point of view these data should be usefully employed only as starting points for more detailed studies.

Table 3 lists the plants already employed in Campania at the beginning of XIX Century, which are still in use. Some species have been used for multiple purposes, including those related to food, medicine, ceremonies, and magic rituals. According to Turner [30], "the more widely or intensively a plant is used, the greater its cultural significance". If we take this assumption as a general rule, it is possible to derive from Table 3 a rank of significance for each plant included, although in a preliminary and qualitative way. In this respect, it is not surprising that the cultural importance of some species as *Olea europea *L. and *Vitis vinifera *L., followed by *Brassica oleracea *L. ranked highest in usage, and consequently in cultural significance, in the Campanian territory. The domestication and cultivation of these species was of pivotal importance in the Mediterranean civilization since Neolithic times, and represent still now an important economic resource for the Campania and the whole Mediterranean Region. The treatment of a range of internal affections with the so called food medicine often relies upon cultivated plants; in general, the collection of wild greens, although still practiced, has been considerably reduced in present times. This is probably due to the continuous decrease of people able to recognize plants in the wild, which, in turn, has been determined by the decline of agriculture in the region. However, cultivated plants correspond to about 30% of the species recorded in Table 3, the remaining being wild species, and it is possible to infer some degree of significance also for these species. For a detailed comparison, we selected three wild gathered species, *Adiantum capillus-veneris *L., *Parietaria judaica *L., and *Artemisia absinthium *L. frequently used in Campania (Table 4).

**Table 3:** Plants used in Campanian folk therapy at the beginning of XIX Century, which are still in use.

**Species**	**Medicinal**	**Food**	**Domestic**	**Ceremonial**	**Veterinary**
*Adiantum capillus-veneris *L.	●				
*Ajuga reptans *L.	●				
*Allium cepa *L. *	●	●			
*Allium sativum *L. *	●	●		●	
*Apium graveolens *L.		●			
*Apium nodiflorum *(L.) Lag.		●			
*Artemisia absinthium *L.	●			●	
*Asparagus acutifolius *L.		●			
*Asparagus officinalis *L. *	●	●			
*Brassica oleracea *L. *	●	●			●
*Brassica rapa *L. s.l. *	●	●			
*Chamaerops humilis *L. *			●	●	
*Cichorium intybus *L. s.l.	●	●			
*Clematis flammula *L.		●			●
*Clematis vitalba *L.		●			
*Castanea sativa *Mill. *		●	●		
*Colchicum autumnale *L. *	●	●			
*Conium maculatum *L.	●				
*Cyclamen hederifolium *Aiton	●				●
*Cydonia oblonga *Mill. *	●	●			
*Cynodon dactylon *(L.) Pers.					●
*Datura stramonium *L. S.l.	●				
*Dianthus *sp.	●				
*Diplotaxis tenuifolia *(L.) DC.		●			
*Erica arborea *L.			●	●	
*Euonymus europaeus *L.	●				
*Euphorbia helioscopia *L. s.l.	●				
*Festuca arundinacea *Schreb. subsp. *corsica *(Hoek.) Kerguélen		●			
*Fragaria vesca *L. s.l.	●	●			
*Fraxinus ornus *L. s.l.	●	●			
*Fumaria capreolata *L. s.l.	●	●			
*Fumaria officinalis *L. s.l.	●	●			
*Hordeum vulgare *L. *	●	●			
*Juglans regia *L. *	●	●	●	●	●
*Lactuca sativa *L. *	●	●			
*Lavandula angustifolia *Mill. s.l.	●		●		
*Malus domestica *(Borkh.) Borkh. *	●	●			
*Matricaria chamomilla *L.	●				
*Mentha *sp.	●	●			
*Mentha *× *piperita *L. *	●	●			
*Nerium oleander *L. s.l. *	●				
*Olea europaea *L. *	●	●		●	
*Origanum majorana *L. *	●	●			
*Origanum vulgare *L. S.l.	●	●			
*Papaver rhoeas *L. s.l.	●				
*Parietaria judaica *L.	●	●			
*Persicaria hydropiper *(L.) Delarbre	●	●			
*Petroselinum crispum *(Mill.) Fuss *	●	●			
*Pimpinella anisum *L. *	●	●			
*Plantago major *L. s.l.	●				
*Portulaca oleracea *L. s.l.		●			
*Quercus *sp.	●				
*Rubus *sp.	●	●		●	
*Ruscus aculeatus *L.	●	●	●	●	●
*Ruta *sp.	●				
*Ruta graveolens *L. *	●				
*Salix alba *L.	●				
*Sambucus nigra *L.	●	●			
*Sanguisorba minor *Scop. s.l.		●			
*Sedum telephium *L. s.l.	●				
*Sonchus oleraceus *L.		●			
*Sonchus tenerrimus *L.		●			
*Stellaria media *(L.) Vill. s.l.	●				
*Teucrium chamaedrys *L. s.l.	●				
*Trifolium incarnatum *L. s.l.					●
*Triticum aestivum *L. *	●	●			
*Typha latifolia *L.			●		
*Verbena *sp.	●				
*Verbena officinalis *L.	●				
*Vitis vinifera *L. *	●	●	●		●

(*) Cultivated plants.

**Table 4:** Campanian ethnopharmacological uses of *Adiantum capillus-veneris *L., *Artemisia absinthium *L. and *Parietaria judaica *L. in the last two centuries.

**Botanical name**	**Uses description**	**Part used**	**Preparations**	**Authors**
*Adiantum capillus-veneris *L.	Against labour pains	Aerial part	Decoction	Cirelli, 1853 [35]
	
	Emmenagogue	Aerial part	Infusion	Limoncelli, 1862 [28]
	
	Local application on sores	Leaves	External use	Gusumpaur, 1887 [36]
	
	Respiratory system affections: emollient, expectorant	Aerial part	Infusion	Antonone et al., 1988 [37]
	
	Resolvent for contusions, antiecchymotic	Aerial part	Decoction	De Feo et al., 1992 [38]
	
	Antitussive, for sore-throat and loss of speech. Externally against hair loss and dandruff	Aerial part	Infusion	De Feo, Senatore, 1993 [39]
	
	Abortive, regulator of menses, anti-cough	Aerial part	Decoction of leaves with some dried figs, an apple, some *Prunus cerasus *L. fruits, some pieces of *Prunus dulcis *(Mill.) D.A. Webb epicarpus	De Natale, Pollio, 2007 [40]
	
	Expectorant, antitussive, in the treatment of bronchitis, against dandruff and seborrhea	Aerial part	Decoction, infusion and tincture	La Palometa, Grieco, 2003 [41]
	
	Antihelmintic	Aerial part	Decoction	De Natale, Pollio, (unpubl.)

*Artemisia absinthium *L.	Against malaria, abdominal pains, antihelmintic (external use)	Aerial part	Decotion (internal use), poultice (external use)	Cirelli, 1853 [35]
	
	Against malaria	Aerial part	External use: poultice with *Allium *sp., *Ruta *sp., *Mentha *sp. on the wrist	Jamalio, 1918 [42]
	
	Abdominal pains	Aerial part	Potion with rue and minth	
	
	1. treatment of diabetes and hypercholesterolemia; 2. stimulate liver function, against biliary calculosis and dyspepsia; 3. external use as cicatrizant for wound and sores; 4. to cure mumps	Aerial part	1. decoction; 2. infusion; 3. topic use (crushed leaves); 4. topic use (maceration in olive oil)	De Feo et al., 1992[43]; De Feo, Senatore, 1993 [39]
	
	Antihelmintic	Aerial part	Infusion with *Ruta *sp., *Mentha *sp., *Matricaria chamomilla *L.	De Natale, Pollio, (unpubl.)

*Parietaria judaica *L.	Treatment of pneumonia (antidolorific, external use)	Aerial part	poultice mixed with *Cicuta virosa *L.	Cirelli, 1853 [35]
	
	Against rhagads (external use)	Aerial part	Poultice	Cavara, 1954 [44]
	
	1. cholagogue and to treat cystitis and in renal and biliary lithiasis; 2. externally to treat sprains and haematomas; 3. fresh plant rubbed on the part to treat nettle rashes and insect bites	Aerial part	1. infusion; 2. decoction; 3. fresh plant	De Feo et al., 1992 [43]
	
	Diuretic, depurative, hemorrhoid lenitive, vermifuge, antitussive, antiecchymotic, resolvent for skin inflammation, sedative in cases of intestinal colic	Aerial part	Infusion, local application, crushed	De Feo et al., 1992 [38]
	
	1. Diuretic digestive refresher, mild laxative; 2. externally, to allay subcutaneous bleeding	Aerial part	1. Infusion; 2. decoction	De Feo, Senatore, 1993 [39]
	
	Diuretic, to cure renal diseases	Aerial part	Decoction	De Natale, Pollio, (unpubl.)
	
	Antiinflammatory, resolutive, for urinal tract. Against cystitis, the decoction of the fresh plant has to be drunk regularly (after a break of some weeks the cure should be repeated).	Aerial part	Decoction	Scherrer et al., 2005 [45]
	Gastrointestinal inflammations and colitis	Aerial part	Local application	

It is evident that even though the general significance of these plants, based on the number of usages, remained unmodified, many present uses differ from those of XIX Century. The analysis of data suggests that in XIX, and in the first decade of the following Century, the selected plants were primarily used as pain-killer, and as febrifuge, whereas their reliance for these scopes is now less frequently reported [31-41]. The capillary spread of conventional pharmaceuticals products, comparatively safer and more efficacious, has probably caused the disappearance of this medicinal use. On the other hand, some new applications probably borrowed from health public media, have been introduced, as indicated by the application of some plants against metabolic diseases, such as high blood pressure, and hypercholesterolemia.

### Extension of the temporal context to the Antiquity

The Campanian historical database encompasses also information on plant uses in ancient Mediterranen cultures, particularly of Greek and Roman phytotherapy. This inclusion needs some methodological considerations: in a straightforward assay, the anthropologist Di Nola [42] categorized different kinds of ethnomedicine. The first one fits the Hoppal [43] definition: "ethnomedicine is the term for the practice of folk healing in the recent ethnographic literature". This definition embraces not only the study of cultures lacking written traditions, but also the body of traditional knowledge of European rural communities. According to Di Nola [42] also the corpus of knowledge belonging to the Ancient Classic medicine can be considered as a sort of ethnomedicine. It consists of treatises where popular phyto-remedies are described, although often tightly intermingled with a scholar approach to the therapy of some diseases. It cannot be denied that a thread linking Ancient and contemporary ethnobotany in Mediterranean Countries can be ideally traced: Stannard [44] stressed that not few curative practices elaborated in early times of Western civilization had remained alive. On the other hand, the caution evoked for the evaluation of ethnobotanical data coming from the last two centuries must be multiplied when one aims to analyze different uses of plants over a so large temporal period.

Paul [45] suggested that popular therapeutics is prevalently made by old medical prescriptions, which were subsequently abandoned by official Medicine. To shed light on this point, the medicinal uses of *A. capillus-veneris*, *A. absinthium *and *P. judaica *have been reported in table 5, according to some of the most important medical treatises of Antiquity and to a medical text of XVIII Century written by V. Petagna [27]. In this way, it is possible to trace the possible influence on present popular medicine of historical recipes from different periods. As can be seen, during Antiquity *A. capillus-veneris *was administred mainly against pulmonar and kidney affections, and to treat gynecological diseases, *A. absinthium *was highly reputed as a tonic and digestive, and as a powerful antiworming agent and *P. judaica *had application as a diuretic, in the therapy of skin affections and to help the resolution of traumatic accidents. Similar therapeutic uses are also descripted by Petagna [27]. A comparison with the present ethnopharmacological knowledge in Campania indicate that only some of the oldest applications are still in use, and that, as expected, there are more similarities between the indications reported by Petagna [27] and the present uses of the selected plants. However, no general rule can be traced; the story of uses of each plant follows hardly identifiable trajectories, which need a detailed study of all available sources. A point break is the transition from Antiquity to the Christian Era, when new symbolic meanings were introduced for many plants, renforcing old therapeutical applications or also relating them to unexplored medical applications, as in the case of *Rosmarinus officinalis *in Latin America [6].

**Table 5:** Campanian ethnopharmacological uses of *Adiantum capillus-veneris *L., *Artemisia absinthium *L. and *Parietaria judaica *L. in medical treatises writtten in different historical periods.

	**Affection**	**Part(s) of plant**	**Preparation**	**Occurrence in XIX-XXI century TK of Campania**
***Adiantum capillus-veneris *L.**				
*Corpus Hippocraticum*	diuretic	aerial part	decoction	
	
	jaundice	aerial part	potion	
	
	to promote menses	aerial part	potion	○
	
	uterine affection	aerial part	potion	
	
	against leucorrhaea	aerial part	potion with wine	
	
	rectum prolapsus	aerial part	local application	
	
	to relieve partum complaints	aerial part	potion	
	to easy placenta expulsion	aerial part	potion	
	
	to facilitate the conception	aerial part	local fumigation	

*Dioscorides*	against asthma	aerial part	decoction	○
	
	jaundice	aerial part	decoction	
	
	mycosis	aerial part	decoction	
	lithiasis and related affections	aerial part	decoction	
	
	against diarrhoea	aerial part	decoction	
	gynecological affections	aerial part	potion with wine	○
	
	emmenagogue	aerial part	potion with wine	○
	against hair losses	aerial part	local application, also in mixture with laudanum, hyssop and honey	●
	against alopaecia	aerial part	local application	●
	
	skin affections	aerial part	local application	●

*Pliny*	affections of the liver and spleen	aerial part	decoction	
	
	against asthma	aerial part	decoction	
	
	against excoriations of infants	aerial part	liniment combined with rose-oil, local application	
	
	against head-ache	aerial part	local application	
	
	antidote to the venom of serpents and spiders	aerial part	local application	
	anti-hæmorrhagic	aerial part	potion with vinegar	
	
	calculi of the bladder	aerial part	potion	
	
	diuretic	aerial part	potion with wine	
	expulsion of after-birth, emmenagogue	aerial part	potion	○
	
	looseness of the bowels	aerial part	decoction in wine	
	
	skin affections	aerial part	local application	○
	strangury and affections of the kidneys	aerial part	liniment, local application	

*Pseudo Apuleius*	abdominal pain	leaves	wine potion with coriander and pepper seeds	○

*Petagna*	respiratory system affections	aerial part	infusion, sirup	●

***Artemisia absinthium *L.**				
*Corpus Hippocraticum*	against sterility	aerial part	not described	
	
	emmenagogue	aerial part	local application, macerated in white wine	
	
	jaundice	aerial part	not described	
	
	opistotonus (neck rigidity)	aerial part	not described	
	
	post partum affections	aerial part	infusion	
	uterine affections	aerial part	local application	

*Dioscorides*	against animal bites or poisonous plants	aerial part	wine potion	
	
	against mosquito bites	aerial part	poultice mixed with oil	
	
	dropsy and spleen pains	aerial part	poultice with Cyprus ointment	
	ear and tooth pains	aerial part	decoction	
	eye affections	aerial part	local application: the plant mixed with oil or honey	
	jaundice, inappetence	aerial part	decoction	
	
	sore throat	aerial part	ointment with figs soda and rye-grass flour	
	
	stomach and visceral pains	aerial part	poultice (topic application) or potion	
	
	tonic	aerial part	wine potion	

*Pliny*	antidote to animal and plant poisons	aerial part	potion with vinegar or wine	
	
	antihelmintic	aerial part	potion of the extracted juice	●
	diuretic	aerial part	potion with gallic nard	
	
	ear affections	aerial part	decoction, local application or liniment of the plant bruised with honey	●
	
	emmenagogue	aerial part	taken with honey or employed as a pessary	
	
	eye affections	aerial part	local application, with raisin wine or honey	
	jaundice	aerial part	eaten raw, with parsley or adiantum	
	liver complaints	aerial part	potion with gallic nard	○
	iliac regions affection	aerial part	plaster with Cyprian wax or figs	
	
	purgative	seeds	potion with sea water and honey	
	sea sickness	aerial part	potion drunken as a preventive	
	skin affections	aerial part	water infusion	
	spleen diseases	aerial part	potion with vinegar and figs	
	stomach affections	aerial part	decoction, or raw with rue, pepper, and salt	
	
	to dispel nausea and flatulency	aerial part	potion with the addition of sile, gallic narde and a little vinegar	
	tonic	aerial part	decoction	○
	wounds	aerial part	local application	●

*Pseudo Apuleius*	not reported	----	--------	

*Petagna*	hypochondria, hysteria	leaves	extract through the soap	○
	
	To stimulate liver function	leaves	extract through the soap	○
	
	against malaria	----	1/2 spoon of juice of the plant with clove in wine	●
	
	against scurvy	leaves	infusion	
	
	against dropsy	leaves	infusion	
	
	antihelmintic	leaves	1 spoon of essence	●
	
	headache		Powdered leaves mixed with sugar, or infusion of diluted wine	
	
	eyes inflammation		powder of leaves mixed with sugar, or infusion of diluted wine	●
	
	edemata	leaves	fresh leaves	
	
	gangrene	leaves	boiled in the sea water	

***Parietaria judaica *L.**				
*Corpus Hippocraticum*	abortive	leaves	potion	

*Dioscorides*	skin affection	leaves	local application	●
	
	glandular inflammation	leaves	local application	
	
	oedema	leaves	local application	
	
	ulcerations	leaves	juice and white lead ointment	
	
	sore throat	leaves	ointment or juice gargle	
	
	earache	leaves	ointment with rose oil	
	
	cough	leaves	juice	

*Pliny*	for suppurated abscesses	leaves	the juice taken warm	
	
	to cure convulsions	leaves	the juice taken warm	
	
	to cure ruptures, bruises	leaves	the juice taken warm	●

*Pseudo Apuleius*	gout	aerial part	decoction mixed with pork fat and applied on foots or knee	

*Petagna*	emollient	leaves	juice, decoction with bearberry	
	
	resolvent	leaves	juice, decoction with bearberry	○
	
	diuretic	leaves	juice, decoction with bearberry	○

The black dots indicate the same medicinal aims use while the white dots similar use.

In this respect, the comparison of the TK of a restricted territory as that of Campania with the corpus of Ancient phytotherapy can not be exhaustive and needs a more detailed scrutiny carried out on a larger set of historical data from other territories of the Mediterranean region.

## Conclusion

The Mediterranean region, with its complex history, still needs historical studies dealing on traditional knowledge of plants. This database revealed to be a useful tool to store and to compare present and previous ethnobotanical uses in the Campanian region. The collection of data is in progress. Further information could be gathered in small libraries and local archives present almost in an Campanian towns, where it could be possible to find forgotten sources dealing with local traditions.

In the next future it is necessary to finalize the structure of the database for publishing it online, adding multilingual support and administration of user privileges, in order to be contributed by other researchers, thus enhancing the Mediterranean ethnobotanical network.

## Competing interests

The authors declare that they have no competing interests.

## Authors' contributions

ADN was involved in setting up the historical database and data analysis. GBP was involved in the design and implementation of the relational database. AP was involved in data collection, archiviation from ancient sources and data analysis.
